# Role of Dibenzo Crown Additive for Improving the Stability of Inorganic Perovskite Solar Cells

**DOI:** 10.3390/nano13111751

**Published:** 2023-05-27

**Authors:** Miao He, Xinyu Xu, Le Zhang, Fei Lu, Chuwu Xing, Duofa Wang, Tianjin Zhang

**Affiliations:** Key Laboratory of Green Preparation and Applicationfor Functional Materials, Ministry of Education, Collaborative Innovation Center for Advanced Organic Chemical Materials Co-Constructed by the Province and Ministry, Hubei Key Laboratory of Polymer Materials, School of Materials Science and Engineering, Hubei University, Wuhan 430062, China; 20110029@hubu.edu.cn (M.H.); 101213847@hubu.edu.cn (F.L.);

**Keywords:** perovskite layer, CsPbI_2_Br, organic doping dynamics, DC additive, photovoltaic

## Abstract

Photovoltaics are being transformed by perovskite solar cells. The power conversion efficiency of these solar cells has increased significantly, and even higher efficiencies are possible. The scientific community has gained much attention due to perovskites’ potential. Herein, the electron-only devices were prepared by spin-coating and introducing the organic molecule dibenzo-18-crown-6 (DC) to CsPbI_2_Br perovskite precursor solution. The current-voltage (I-V) and J-V curves were measured. The morphologies and elemental composition information of the samples were obtained by SEM, XRD, XPS, Raman, and photoluminescence (PL) spectroscopies. The distinct impact of organic DC molecules on the phase, morphology, and optical properties of perovskite films are examined and interpreted with experimental results. The efficiency of the photovoltaic device in the control group is 9.76%, and the device efficiency gradually increases with the increase of DC concentration. When the concentration is 0.3%, the device efficiency is the best, reaching 11.57%, short-circuit current is 14.01 mA/cm^2^, the open circuit voltage is 1.19 V, and the fill factor is 0.7. The presence of DC molecules effectively controlled the perovskite crystallization process by inhibiting the in-situ generations of impurity phases and minimizing the defect density of the film.

## 1. Introduction

Perovskite solar cells, a promising new photovoltaic technology, have exhibited a rapid increase in efficiency from 3.8% to 25.7% in the past decade [1], with the incorporation of the most efficient PSCs (PCE > 20%) composed of organic-inorganic hybrid calcium [2,3,4,5]. However, organic-inorganic hybrid perovskite materials have poor stability when exposed to heat, oxygen, moisture, and even light [2,6,7]. Higher intrinsic stability of perovskite materials is achieved when organic cations are replaced by inorganic cesium ions [8]. Inorganic perovskites are not easily decomposed due to the absence of volatiles and the presence of heat, moisture, and hygroscopic organic cations. The preparation process of inorganic perovskites generally requires high-temperature annealing, and a higher fabrication cost, which is not suitable for the commercialization of technology [1,4]. Previously it has been reported that the crystalline quality of inorganic perovskites remains poor at low temperatures along with an impurity of cesium bromide [9]. Although the introduction of the organic molecule can play a certain inhibitory role, the organic molecule interacts with lead iodide in the perovskite and indirectly inhibits the generation of cesium bromide [9,10,11]. Lately, additive engineering is recognized as an efficient technique for the structural optimization of the perovskite films to enlarge crystal grain sizes along with minimized defect densities [10,11]. According to literature reports [5,10], crown ethers and their derivatives can be combined owing to their molecular structure and electronegativity. Here alkali metal [12] ions have a significant role. Studies have shown that the introduction of crown ethers and their derivatives can effectively improve the crystalline quality of perovskite films as well as device performance [13]. Also, the salts containing metal halides offer few ions, such as Co^2+^, Rb^+^, and Cs^+^ with comparable radii for the partial substitution of the M^+^ or Pb^2+^ to modify the Gold-Schmidt tolerance factor for the creation of the efficient perovskite crystal structure equipped with excellent morphological and charge transport characteristics [14]. Moreover, the incorporation of some molecules/polymers can directly passivate the defects through unique intermolecular bonding interactions in the perovskite film. Interestingly, there are several molecules/polymers (i.e., PCBM, ITIC, and PBDB-T) [15] extensively studied for their exclusive hydrogen bond interaction with the Lewis base group for effective removal of the PbI anti-site defects at grain boundaries. Polymers with a long chain of multiple functional groups exhibit stable interactions as compared to small molecules in cross-linking the perovskite grain boundaries thus offering induced stability in the film [16]. Similarly, various polymers such as polyacrylic acid (PAA), polyethylene glycol (PEG), polyethyleneimine (b-PEI), and poly (4-vinyl pyridine) (PVP) have been incorporated for good results [14,16].

**Figure 1:**
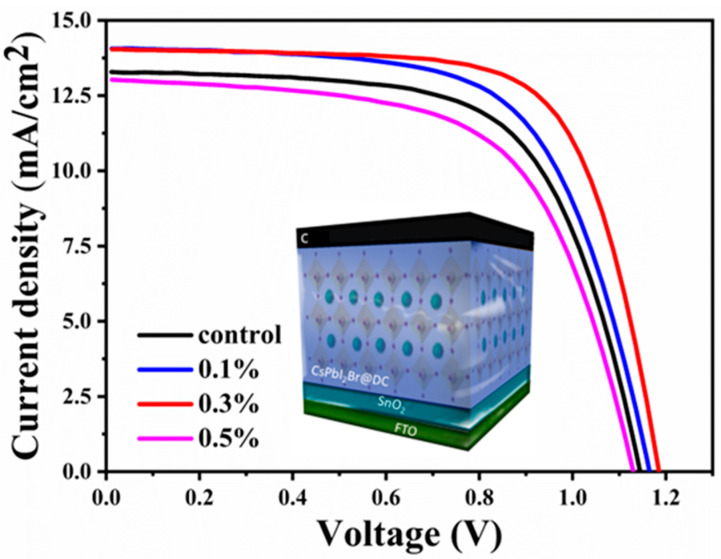
Schematic illustration of doping dynamics of dibenzo crown as an efficient additive for improving the efficiency and stability of CsPbI_2_Br-based perovskite solar cells.

The focus of this research work is mainly to introduce a new organic molecule as an additive to fine-tune the crystallization process of perovskite films after a direct interaction with cesium ions at low temperature (130 °C). In this work, all-inorganic perovskite high-efficiency solar cell devices are prepared by introducing the organic molecule dibenzo-18-crown-6 (DC) into the precursor solution, due to its molecular structure and electronegativity, crown ether and its derivatives can interact with alkali metal ions (DC is one kind of crown ether derivatives). The concentration of DC molecules was 0.0%, 0.1%, 0.3%, and 0.5%. The sample doped with 0.0% organic DC molecules was named as a control group and the samples doped with 0.1%, 0.3%, and 0.5% were named as the experimental group. The as-prepared perovskite material is characterized for structure, surface, luminesce, and composition through the XRD, SEM, PL, XPS, and infrared techniques respectively. The distinct impact of organic DC molecules on the phase, morphology, and optical properties of perovskite films are examined and interpreted with experimental results. The temperature-dependent transformation of the CsPbI_2_Br perovskite layer via organic molecule diben-zo-18-crown-6 (DC) to CsPbI_2_Br perovskite precursor solution, resulting into a significant in-crease solar cell efficiency, as illustrated in Figure 1. The presence of DC molecules effectively controlled the perovskite crystallization process by inhibiting the in-situ generations of impurity phases and minimizing the defect density of the film.

## 2. Materials and Methods

### 2.1. Materials

Lead bromide (PbBr_2_) (≥99.999%), and cesium iodide (CsI) (≥99.999%) were purchased from the Aladdin Chemical Reagent Co., Ltd., Shanghai, China. Whereas, N-N dimethyl cool glue (C_3_H_2_NO) (99.8%), Dimethyl sulfoxide (C_2_H_6_OS) (99.9%), and Chlorobenzene (C_6_H_5_Cl) were obtained from Sigma Aldrich Co., Ltd., Beijing, China. C_20_H_24_O_6_ and SnO_2_ (15% in water) were offered from Alfa Aesar Chemicals Co., Ltd., Shanghai, China. While, C_3_COCH_3_, C_2_H_6_O, and C_3_H_8_O were bought from Shanghai Sinopharm Co., Ltd., Shanghai, China. C_72_H_14_O_2_ (99.9%) and PbI_2_ (99.9%) were obtained from the Xian Baolaite Photoelectric Technology Co., Ltd., Xi’an, Shaanxi, China. Company. Au was offered by Beijing Hezhong New Material Co., Ltd., Beijing, China. While, carbon slurry C was taken from Shanghai MaterWin New Materials Co., Ltd., Shanghai, China. All the obtained materials were sustaining a 99% purity level and were processed without further purification.

### 2.2. SnO_2_ thin Films Preparation

First of all, the pieces of FTO glass (size 2 cm × 2 cm) were cleaned with deionized water, acetone, isopropanol, and ethanol for 30 min via ultrasonic cleaning, and then dried completely using nitrogen gas. After that, the SnO_2_ transport layer was deposited on FTO glasses. For this, SnO_2_ colloidal solution was prepared in deionized water at a volume ratio of 1:3 under constant stirring and allowed to get uniform dispersion. The cleaned FTO substrates were ozonated for 20 min in a UV Ozonator. The as-prepared solution of SnO_2_ was deposited on the FTO substrates via the spin-coating method, with a rotation speed of 4000 r/min and acceleration of 2000 rpm/s for 30 s. After spin coating, the film was annealed at 150 °C for 30 min.

### 2.3. Perovskite Film Fabrication

The precursor solutions with the molar ratio 2∶1∶1 of CsI, PbI_2_, and PbBr_2_ and the volume ratio of 89∶35 of DMF to DMSO were prepared respectively. For devices with DC, different proportions (0.1%, 0.3%, 0.5%) of DC were added to perovskite precursor solution for preparation An appropriate amount of solute was added to the mixed solvent and the precursor concentration was 0.8 mol/L. Then precursor solutions were prepared with different concentrations of additives. The molar ratios of different concentrations of additives to CsPbI_2_Br were different. Before spin coating, the FTO/SnO_2_ coated substrates were ozone treated in the UV ozone machine for 20 min. The prepared precursor solution was dropped on FTO/SnO_2_ substrates using a spin coater which was rotated at the speed of 4000 rpm/s for 30 s. Finally, the annealing was carried out in two steps: the annealing was performed at 100 °C for 5 min and 130 °C for 30 min. 

### 2.4. Carbon Electrode Preparation

The conductive carbon paste was scraped onto the FTO/SnO_2_/perovskite film by screen printing method, and then annealed at 130 °C for 15 min in air. Finally, carbon electrode with area of 0.070 cm^2^ was obtained. 

### 2.5. Characterizations

The morphologies and elemental composition information of the samples were obtained by a JSM-6700F feld emission scanning electron microscopy (Japan Electronics Corporation, Tokyo, Honshu, Japan). X-ray diffraction (XRD) pattern was recorded via a Bruker D8 advance diffractometer with Cu Kα (λ = 1.54178 Å) (Germany). X-ray photoelectron spectroscopy (XPS) and ultraviolet photoemission spectroscopy (UPS) measurements were conducted on a VG ESCALAB MK2 system (Manchester, Britain) with monochromatized Al Karadiation and He l (21.2ev) ultraviolet light source respectively. The photoluminescence (PL) measurements were carried out on a transient spectroscopy system (FluoTime 300, Pico Quant GmbH, Berlin, Germany). Electrochemical impedance spectroscopies (EIS) were carried out with an electrochemical workstation (Zahner Zennium Pro, Zahner, Kronach, Germany) under dark with different biases over the frequency range of 1 Hz to 2 MHz. Current density-voltage (J-V) curves of devices were obtained using a Keithley 2401 source (Keithley, Cleveland, OH, USA) meter under the illumination of AM 1.5 G (100 mW/cm^2^) provided by a 91192-1000 solar simulator (Newport Corporation-Oriel Instruments, Mountain View, CA, USA). The light intensity was corrected by a standard Si reference cell before the test. The area of devices was controlled at 0.070 cm^2^ by a black metal mask. The steady-state output of the photocurrent curves and PCEs were measured with a Keithley 2401 digital source meter under a certain bias. External quantum efficiency (EQE) was recorded on a Keithley 2000 multimeter (Keithley, USA) with the illumination of a 300 W tungsten lamp and a Spectral Product DK240 monochromator (SP, USA).

## 3. Results and Discussion

### 3.1. Crystal Growth and Morphological Analysis

**Figure 2:**
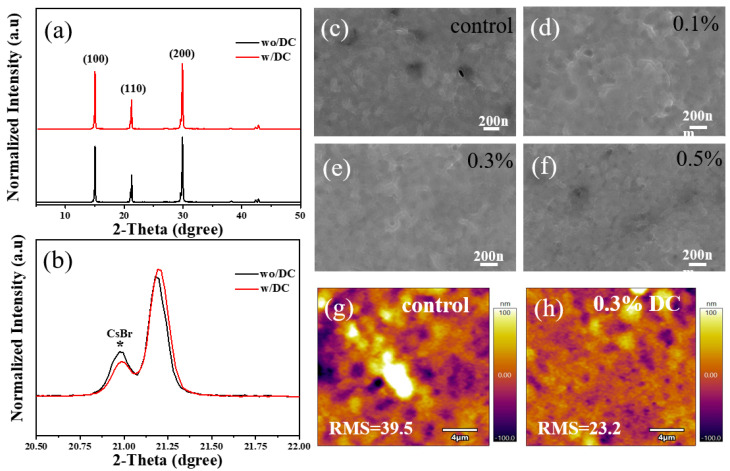
(**a**) XRD patterns of perovskite films in the control and experimental groups. (**b**) Higher magnification XRD patterns around the (110) crystal plane, symbolic ”*” refer to characteristic peak of cesium bromide. (**c**–**f**) SEM images of the surface topography of perovskite films with different additive concentrations. (**g**,**h**) AFM profiles of the control and experimental groups.

To study the effect of organic DC on the microscopic phase of perovskite thin films, XRD tests were carried out by preparing the perovskite film samples of the control group and the experimental group, and the results are shown in Figure 2a,b. From Figure 2a, it can be seen that the characteristic diffraction peaks of the perovskite α phase are in (100), (110), and (200) planes. Whereas, the perovskite film grows along the (100) orientation. The diffraction peak intensities of the samples in the two groups are not much different, and no other diffraction peaks appear, indicating that the introduction of organic molecules did not destroy the crystal structure of perovskite. These results are in good agreement with the results reported in the previous work [17,18,19]. The XRD pattern is partially enlarged as shown in Figure 2b, the diffraction peak of cesium bromide appears at 20.9° and the presence of cesium bromide is suppressed with the introduction of organic DC molecules. SEM was used to observe the surface morphology of the perovskite films in the control group and the experimental group as shown in Figure 2c–f. Figure 1c shows the surface morphology of the film of the control group. It can be seen that there are a lot of pores on the surface of the film. These pores will affect the transport of carriers and have a very negative impact on the performance of the PV device. Figure 2c–f show the surface morphologies of thin films grown with different additive concentrations. With the increase of additive concentration, the pores on the surface of the thin film gradually decreased i.e., the grains are closely arranged and the film grown with an additive concentration of 0.3%, the surface morphology of the film is improved as a proportion of pores are bridged. When the additive concentration exceeds 0.3%, the surface morphology deteriorates again, because too much additive can destroy the crystallization of perovskite material [20,21].

Further AFM measurement was performed on the film samples of the control group and the experimental group, as shown in Figure 2g,h. It can be observed that the surface roughness of the film in the experimental group is significantly lower than that of the film in the control group. In other words, with the increase of additive concentration, the surface of the film becomes smoother and smoother. The smooth surface of the perovskite film is proved to be more conducive and acts as a good transport layer i.e., which can better transport the carriers, thereby improving the device’s performance.

### 3.2. Compositional Moleculer Interaction Analysis

**Figure 3:**
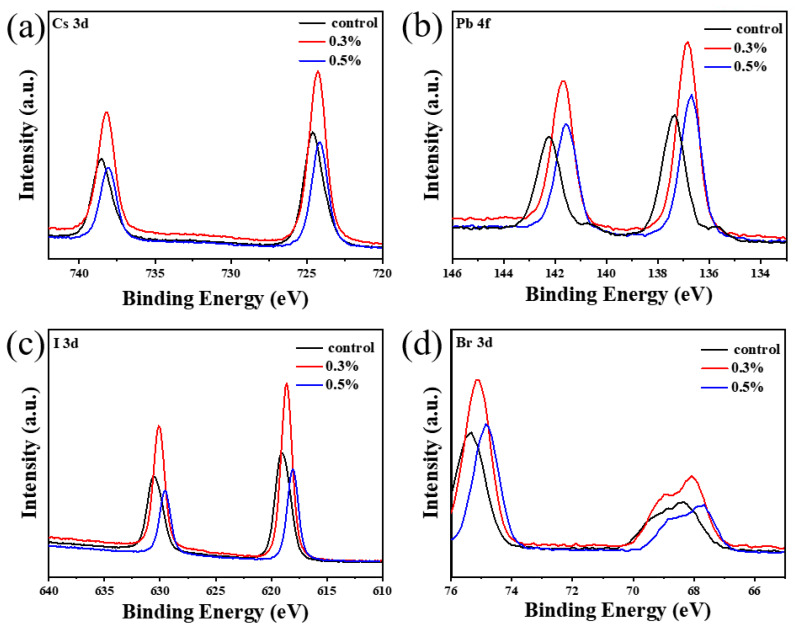
XPS analysis pattern of perovskite film, XPS element analysis of (**a**) Cs element, (**b**) Pb element, (**c**) I element, and (**d**) Br element with 0%, 0.3%, 0.5% DC.

**Figure 4:**
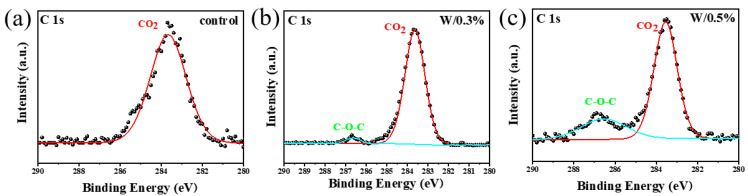
XPS analysis pattern of perovskite film, XPS elemental analysis of (**a**–**c**) C elements with 0%, 0.3%, 0.5% DC.

In order to study the effect of additive DC on the electronic structure of each atom in perovskite films, XPS analysis was performed on perovskite films of control group and experimental group. As shown in Figure 3a–d, unimodal peaks of each element in the film of the experimental group were compared with those in the film of the control group. It can be found that with the increase of DC addition, the peaks of all elements shift towards the direction of smaller binding energy, which indicates that the density of electron cloud around each atom increases, and the bonding ability of each atom in perovskite is stronger, which indicates the interaction between DC and each element in perovskite. Moreover, it is found that the characteristic peak-to-peak intensity reaches the maximum when the addition amount of DC is 0.3%. This is because when 0.3% DC is added, the device has the least defects and the detector collects more optoelectronic signals during the test. In addition, in order to verify the presence of DC molecules in the perovskite film after annealing, XPS was used to test the chemical state of carbon elements in the perovskite structure under different DC addition amounts, as shown in Figure 4a–c. There is only one CO_2_ adsorption peak in Figure 4a. With the increase of DC addition, a new C-O-C peak can be observed at about 286.7 eV, indicating that DC exists in the annealed perovskite film.

### 3.3. Functional Groups Analysis

**Figure 5:**
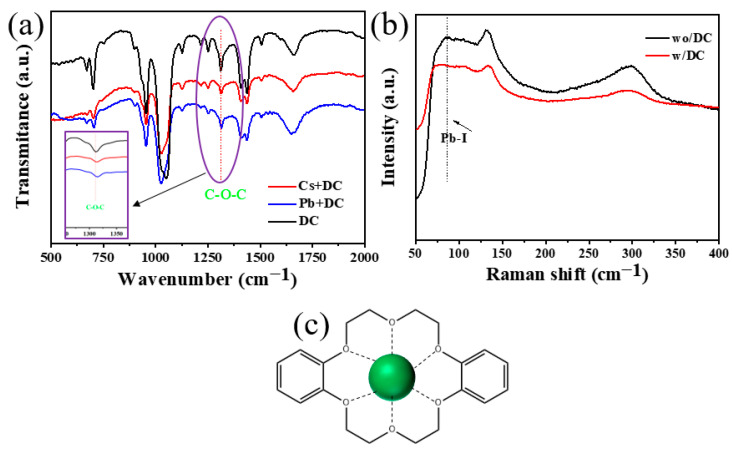
(**a**) FTIR spectra of DC, DC-CsI, and DC-PbI_2_, (**b**) Raman spectra of control and experimental perovskite films, (**c**) schematic diagram of the interaction between DC and cesium ions.

To further explore the interaction between organic DC molecules with cesium ions and lead ions in perovskites. We have prepared the pure DC solution, mixed solution of DC and CsI, and mixed solution of DC and PbI_2_. The obtained Fourier transform infrared spectroscopy results are shown in Figure 5a. In the infrared spectrum of organic DC molecule, the stretching vibration peak of C-O-C in DC appeared from about 1310 to 1315 cm^−1^. It can be seen from Figure 5a that for the mixed solution of DC and CsI and the mixed solution of DC and PbI_2_, the stretching vibration peaks of C-O-C in the spectrum are shifted. The shift of the C-O-C peak position proves that there is an interaction between DC and CsI and PbI_2_, which indicates that DC molecules can interact not only with cesium ions but also with lead ions.

The Raman spectra of the films of the control group and the experimental group are shown in Figure 5b. Compared with the control group, the Raman spectra of the films of the experimental group showed that the tensile vibration peak of PbI from about 86 to 75 cm^−1^ is present, which further confirms that the organic DC molecule interacts with the lead in the perovskite. The schematic diagram in Figure 5c reveals the specific interaction between the additive DC and cesium ions. The pore diameter of DC is 0.26–0.32 nm and the diameter of cesium ions is 0.32 nm, which is very suitable for DC to form Cs + -O bonds for the formation of complexes [22]. The formation of complexes of organic DC molecules with cesium ions affects the perovskite crystallization process. To investigate insight into the formation process of CsPbI_2_Br, the films of the control group and experimental group were prepared in the air without controlling humidity. The prepared films of the control and experimental groups were placed in ambient air (RH: 30%, T: 25 °C) for some time to observe their crystallization process. As shown in Figure 5a, the experimental group films were transparent white, while the control group film turned brown-black after 3 min, indicating that the control group film crystallized rapidly in air and the quality of the film is poor and the surface is rough. Moreover, the perovskite film is completely decomposed after placing one hour in the air, this is mainly due to the influence of water in the air. With the addition of DC, the rapid crystallization process was greatly hindered and the film in the experimental group turned brown-black after 10 min. The control and experimental group films after placing for 10 min in the air were then annealed at 130 °C for 10 min. The control group film was found to be lighter brown, while the DC-optimized film crystallized well, with a brown-black color and a smooth surface. According to the literature, a slower crystallization rate is beneficial to improve the quality of perovskite films [23].

### 3.4. Transformation of δ into α-Phase of CsPbI_2_Br

**Figure 6:**
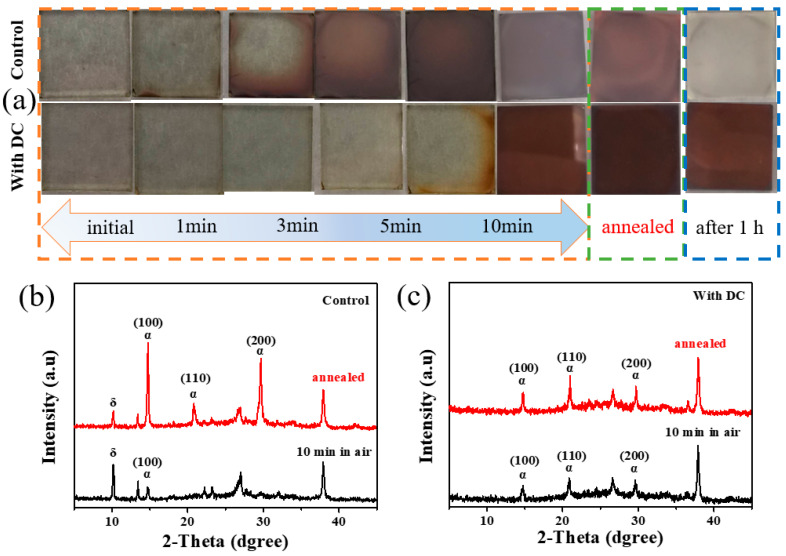
(**a**) Different optical photographs of control and DC-CsPbI_2_Br precursor films stored in ambient air (RH: 30%, T: 25 °C), (**b**) control group (**c**) experimental group CsPbI_2_Br precursor XRD patterns of bulk films stored in ambient air for 10 min and annealed at 130 °C for 10 min after storage.

Different optical photographs of control and DC-CsPbI2Br precursor films stored in ambient air (RH: 30%, T: 25 °C) as demonstarted in Figure 6a. The crystal structure of the control and experimental group films before and after annealing were monitored by XRD patterns as shown in Figure 6b,c. The control group film after placing 10 min in the air has crystallized into a small amount of α-phase CsPbI_2_Br perovskite along with δ-phase perovskite. After annealing at 130 °C for 10 min, the diffraction intensity of α-phase CsPbI_2_Br increased, while the δ-phase decreased, which was due to the partial transformation of δ-phase into α-phase under the effect of annealing temperature. In contrast, the films of the experimental group did not show a delta phase either before or after annealing. Therefore, the DC additive can suppress the air-induced crystallization and phase transition of the precursor, which is beneficial to obtain pure phase (phase-free) perovskite films.

**Figure 7:**
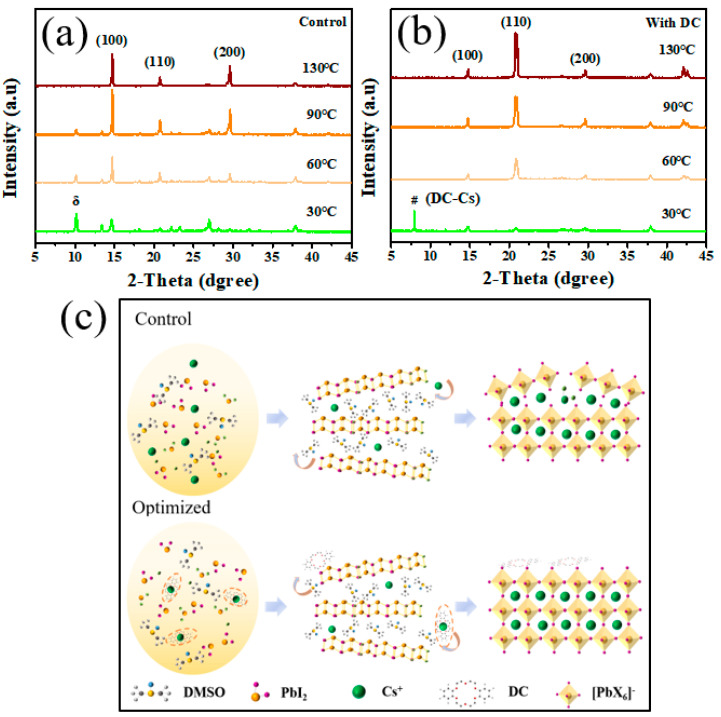
XRD patterns of thin films in (**a**) control and (**b**) experimental groups during annealing, “#” is characteristic peak of precursor complex(DC-Cs). (**c**) Schematic diagram of the crystallization process of the control group and the experimental group, the structure inside the orange oval dotted line represents #(DC-Cs).

To investigate the crystallization process and the phase changes of the control and experimental group films during annealing, XRD characterization was performed, as shown in Figure 7a,b. During the annealing process, the characteristic diffraction peaks of the perovskite δ phase were observed in the control group film. These diffraction peaks of the perovskite δ phase gradually decrease with the increase in temperature and disappeared until 130 °C, indicating that the control group film could not inhibit the formation of moisture in the air during the annealing process [23]. The induced destruction of the perovskite exhibits an unfavorable transition phase [20].

For the experimental group films containing DC additives, the low-angle diffraction peaks of the low-dimensional mesophase were observed at the initial stage of annealing, and the δ phase did not appear. This indicates that the crystallization process of the DC-optimized film was from the mesophase to the α phase. In other words, during the transition process, no unfavorable phase appeared in the experimental group films like in the control group film based on the above experimental results and the results of the earlier study [24] on the perovskite crystallization process, a schematic diagram of the crystallization process of the control and experimental group films is drawn, as shown in Figure 7c. During the crystallization process of the control group film, the reaction of the raw materials was incomplete, resulting in the presence of cesium bromide impurity in the final film, which seriously affected the film quality. For the experimental group films, when forming the DC precursor film, the additive DC, and the formation of complexes by cesium ions slows the rate of cesium ions entering the lead iodide layered structure, thereby slowing down the crystallization process, and also preventing the combination of cesium ions and bromide ions to form insoluble cesium bromide, and finally obtain the high-quality film.

### 3.5. Detailed PV & IV Characteristics Analysis

**Figure 8:**
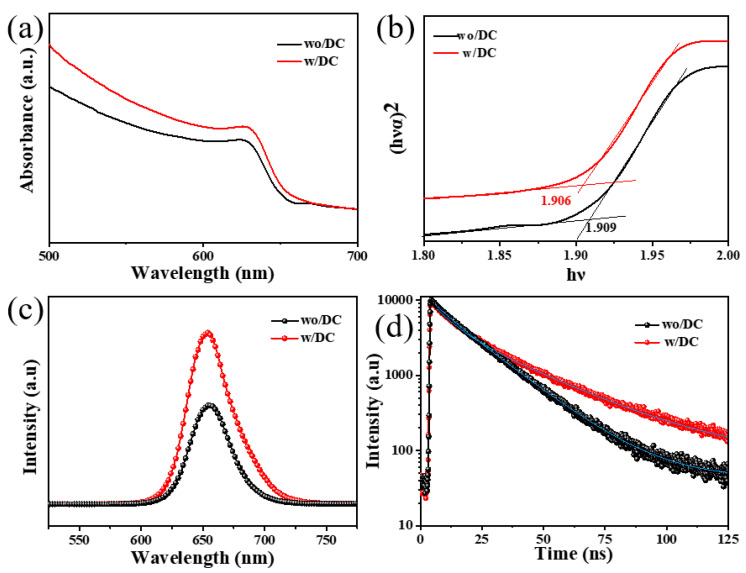
(**a**) UV absorption spectra of the control and experimental perovskite films, (**b**) (hνα)^2^–vs. (hν) curves of the perovskite films, (**c**) PL spectra of perovskite films in control and experimental groups, (**d**) TRPL decay curve.

Figure 8a,b shows the UV-νis absorption spectrum of the control and the experimental group films. From Figure 8a, it can be seen that the absorption intensity of the film in the experimental group has increased compared with the control group film. It is mainly due to the improvement of the crystallinity of perovskite films. To calculate the band gap, the UV absorption spectrum was converted according to the Kubelka-MunK formula [25], and the relationship between (Ahν)^2^ and hν is shown in Figure 8b. The calculated band gap of the control group CsPbI2Br film was 1.909 eV, and the band gap of the experimental group CsPbI_2_Br film was 1.906 eV. The change in the band gap of the control and the experimental group films is almost negligible, indicating that the introduction of additives does not affect the band gap of perovskite materials. The effect of additive DC molecules on the defect density in perovskite films was qualitatively analyzed by directly spin-coating the perovskite layer on the quartz glass substrate. The PL and TRPL tests were performed on the control and experimental group perovskite films, as shown in Figure 8c,d. The TRPL fitting results in Figure 8d showed that the average life time increased from 16.21 to 23.06 ns after adding DC.

From Figure 8c, it can be found that the fluorescence peak intensity of the film in the experimental group is greater compared with the film in the control group, indicating that the film in the experimental group has less non-radiative recombination behavior and less energy loss, so the intensity is stronger. This also means that the defect states in the film of the experimental group are less as compared with the control group film. Therefore, the defect will become a non-radiative recombination center, trapping carriers and causing energy loss. Correspondingly, the results of the TRPL test also reflect the longer carrier lifetime in the films of the experimental group compared with the film in the control group, which further indicates that there are fewer defect states in the films of the experimental group, and the non-radiative recombination is reduced (Figure 8d). As consequence, the presence of the additive DC can eliminate film defects, resulting in higher-quality perovskite films.

**Figure 9:**
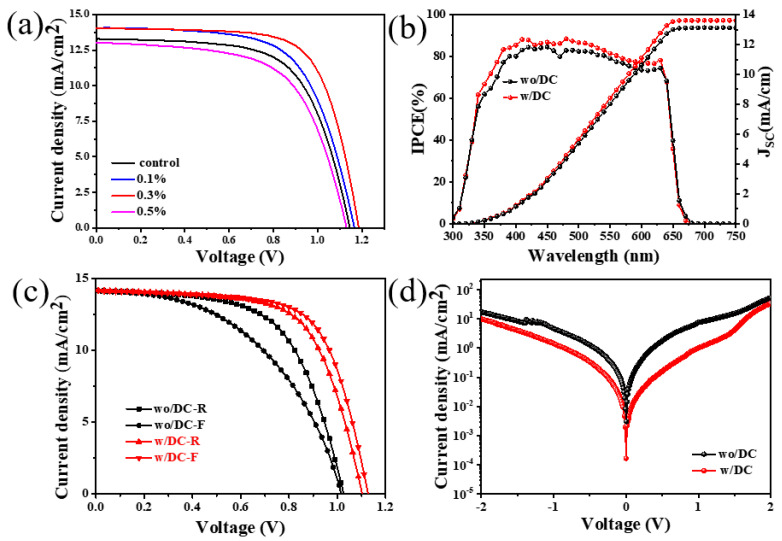
(**a**) The device performance map for different additive concentrations, (**b**) the external quantum efficiency map of the control and experimental films. (**c**) J-V curves of forward and reverse sweeps of devices in the control group and experimental group, (**d**) dark current diagram.

To study the effect of additive DC on the performance of perovskite solar cells, we have measured the efficiency and external quantum efficiency of the control and the experimental group films-based devices as illustrated in Figure 9a. The performance parameters of devices calculated from J-V curves are listed in Table 1 and Table 2. It can be seen that the efficiency of the device made with the film of the control group is 9.76%, and the device efficiency gradually increases with the increase of DC concentration. When the concentration of DC is 0.3%, the device efficiency is the best, reaching 11.57%, with a short-circuit current of 14.01 mA/cm^2^, open circuit voltage of 1.19 V, and a fill factor of 0.7. This is mainly because of the presence of DC [26,27,28], which delays the perovskite crystallization process, reduces the defect density of the perovskite film, and improves the film quality. But when the additive concentration exceeds 0.3%, the device performance begins to decrease because too many organic molecules affect the device’s electrical conductivity [29,30,31]. Figure 9b delineates the external quantum efficiency (EQE). According to the value of EQE, the current of the photovoltaic device is increased. It can be seen from the figure that the light absorption ranges of the film after introducing the additive have not much changed, but the EQE value has increased indicating that the light absorption capacity and current of the perovskite film optimized with the additive DC increase. The forward and reverse currents of the devices in the control group and the experimental group were measured as shown in Figure 9c, and the performance parameters of various devices are listed in Table 2. It can be seen that the reverse current of the device optimized with DC additive (experimental group) is reduced. The main reasons for the reverse current reduction are ion migration and defects. Figure 9d shows the dark current measurements of the control group and the experimental group films-based devices. The results also show that the dark current of the DC-optimized device is a lower order of magnitude than that of the control group device, which further demonstrates that the additive DC can effectively reduce the defect density of the films.

**Figure 10:**
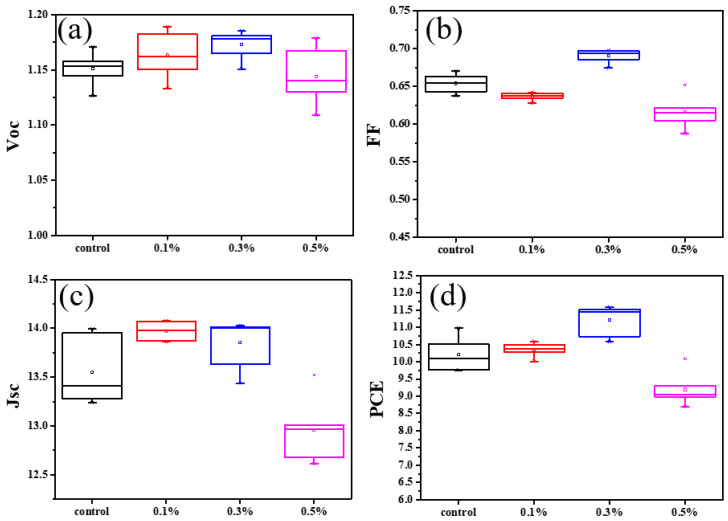
Distribution of performance parameters of perovskite cell devices with different additive concentrations, the statistics of open circuit voltage (**a**), filling factor (**b**), short circuit current (**c**), and photoelectric conversion efficiency (**d**).

The repeatability of perovskite cell devices is judged by testing the performance parameters of multiple PV devices and drawing a statistical graph, as shown in Figure 10a–d, it shows the statistics of open circuit voltage (a), filling factor (b), short circuit current (c), and photoelectric conversion efficiency (d) at different DC additive concentrations. It can be seen that whether it is the control device or the PV device containing additives, the distribution of performance parameters is relatively concentrated, indicating that the used preparation method has good repeatability. It can also be seen that the improvement in the efficiency of the PV device is due to the improved fill factor, which is due to the reduction of the defect density of the film, the reduction of non-radiative recombination, and the improvement of the overall quality of the film under the optimization of additives.

**Figure 11:**
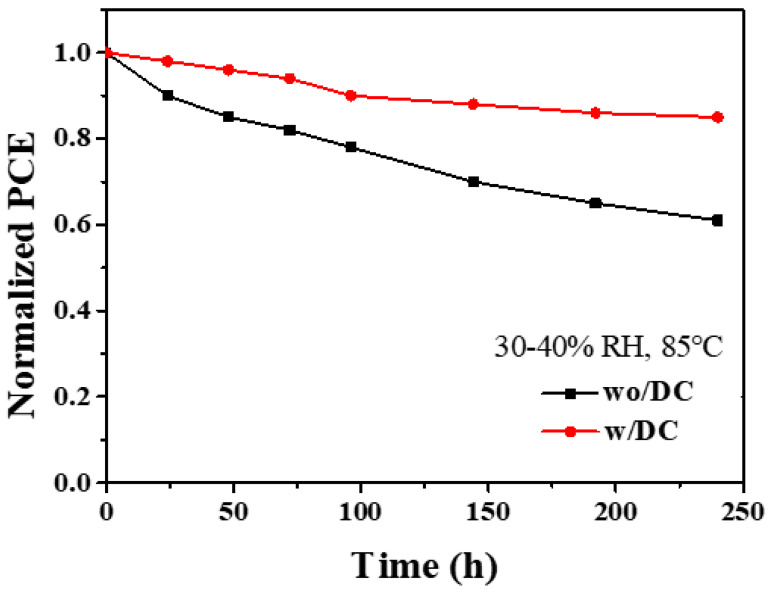
PCE versus time for unencapsulated control and experimental devices on an 85 °C oven (30–40% humidity).

Figure 11 shows the results of PCE versus time for solar cells devices at 85 °C and 30–40% air humidity. It can be seen that the solar cells device optimized by the additive still retains more than 85% of the original efficiency after 240 h, while the efficiency of the un-optimized solar cells device has dropped to 60% of the original efficiency, which indicates that the presence of additives is beneficial to the stability of solar cells devices. The reasons for the improved device performance can be attributed to the following points: Small organic molecules and cesium ions form complexes during the crystallization process of perovskite, inhibit the formation of cesium bromide and δ phase, optimize the crystallization process of perovskite, and thus prepare high quality perovskite thin film. After annealing, DC also combines with uncoordinated Pb, which enhances the stability of perovskite. This can be proved by the peak shift of Pb element in XPS analysis. Through improving the crystallization quality and reducing the defect density, the photoelectric conversion efficiency and the thermal stability of perovskite solar cell devices were increased. We compared the performance parameters of perovskite solar cells with different additives at different preparation temperatures according to literature reports [32], as shown in Table 3. 

## 4. Conclusions

In summary, efficient, and stable organic-inorganic perovskite solar cells were prepared by introducing organic DC molecules into the perovskite precursor solution. The highest efficiency of the device reached 11.57%. After being placed for 240 h under the condition of 30–40% humidity, it can still maintain 85% of the original efficiency and it showed good stability. In-depth research was carried out from the perspective of film phase, action mechanism, and photovoltaic performance, and it was found that the introduction of additives made the film phase pure, the film surface denser, increased the carrier lifetime, and inhibited non-radiative recombination. The influence of the introduction of additives on the phase and surface morphology of the perovskite film was studied by XRD, SEM, and other tests. Due to the interaction between the organic DC molecule and the cesium ion, the heterophase of cesium bromide produced can be effectively inhibited and the surface morphology of the film was improved. The mechanism of interaction between organic molecules DC and perovskite was analyzed by XPS and infrared spectroscopy. Cs+-O bonds were formed between DC and cesium ions to form complexes. The formation of complexes could delay the crystallization process and improve the film quality, reducing the defect density of the film, and finally obtaining a higher-efficiency perovskite solar cell device.

## Figures and Tables

**Table 1:** Performance parameters of perovskite cells with different additive concentrations.

DC%	J_sc_ (mA/cm^2^)	V_oc_ (V)	FF	PCE (%)
0%	13.27	1.14	0.64	9.76
0.1%	14.05	1.14	0.64	10.49
0.3%	14.01	1.19	0.70	11.57
0.5%	12.99	1.13	0.61	9.06

**Table 2:** The forward and reverse scan performance parameters of the control and optimal concentration devices.

Film	J_sc_ (mA/cm^2^)	V_oc_ (V)	FF	PCE (%)
0% DC-reverse	14.15	1.03	0.60	8.75
0% DC-forward	14.13	1.02	0.48	6.96
0.3% DC-reverse	14.17	1.13	0.67	10.75
0.3% DC-forward	14.18	1.10	0.65	10.16

**Table 3:** Performance parameters of perovskite cells with different additive concentrations at different preparation temperatures.

Additive	Device Configuration	Preparation Temperature (°C)	J_SC_ (mA cm^−2^)	V_OC_ (V)	FF	PCE (%)
B site Sr^2+^	FTO/c-TiO_2_/m-TiO_2_/CsPb_0.98_Sr_0.02_I_2_Br/P3HT/Au	310	15.3	1.043	0.699	11.2
B site Cd^2+^	FTO/TiO_2_/CsPbIBr_2_-Cd^2+^/Carbon	160	11.53	1.324	0.696	10.63
B site Gd^3+^	FTO/TiO_2_/CsPbI_2_Br_0.96_ (GdCl_3_)_0.04_/Spiro-OMeTAD/Au	160	16.09	1.222	0.825	16.24
HI	FTO/c-TiO_2_/CsPbI_3_(γ)/P3HT/Au	100	16.53	1.04	0.657	11.3
HPbI_3_	FTO/c-TiO_2_/m-TiO_2_/CsPbI_3_(α)/Carbon	200	18.5	0.79	0.65	9.5
HPbI_3_	FTO/c-TiO_2_/CsPbI_3_/Spiro-OMeTAD/Au	180	18.4	1.054	0.74	14.3
DMAI	FTO/PEDOT:PSS/CsPbI_3_/(C60/BCP)/Ag	100	16.65	0.99	0.765	12.62
HEMA	FTO/ZnO/CsPbI_2_Br/PM6/MoO_3_/Ag	160	15.81	1.23	0.83	16.13
MACl	FTO/c-TiO_2_/CsPbI_3_/spiro-OMeTAD/Au	100	20.59	1.198	0.825	20.37
GuaSCN	FTO/c-TiO_2_/CsPbIBr_2_/Spiro-OMeTAD/Au	280	12.05	1.23	0.737	10.9
DC (this work)	FTO/SnO_2_/CsPbI_2_Br/Carbon	130	14.01	1.19	0.70	11.57

## Data Availability

The data supporting the findings of this study are available from the corresponding authors upon reasonable request.
